# Sepsis Caused by Extended-Spectrum Beta-Lactamase (ESBL)-Positive *K*. *pneumoniae* and *E*. *coli*: Comparison of Severity of Sepsis, Delay of Anti-Infective Therapy and ESBL Genotype

**DOI:** 10.1371/journal.pone.0158039

**Published:** 2016-07-21

**Authors:** Christian Sakellariou, Stephan Gürntke, Ivo Steinmetz, Christian Kohler, Yvonne Pfeifer, Petra Gastmeier, Frank Schwab, Axel Kola, Maria Deja, Rasmus Leistner

**Affiliations:** 1 Institute of Hygiene and Environmental Medicine, National Reference Center for the Surveillance of Nosocomial Infections, Charité Universitaetsmedizin Berlin, Hindenburgdamm 27, 12203 Berlin, Germany; 2 Friedrich Loffler Institute of Medical Microbiology, Universitaetsmedizin Greifswald, Martin-Luther-Str.6, 17475, Greifswald, Germany; 3 Robert Koch Institute, FG13 Nosocomial Pathogens and Antibiotic Resistance, 38855, Wernigerode, Germany; 4 Department of Anesthesiology and Intensive Care, Charité Universitaetsmedizin Berlin, Campus Benjamin Franklin, Hindenburgdamm 30, 12203, Berlin, Germany; The University of Hong Kong, CHINA

## Abstract

Infections with extended-spectrum beta-lactamase-producing *Enterobacteriaceae* (ESBL-E) are associated with increased mortality. Outcome differences due to various species of ESBL-E or ESBL genotypes are not well investigated. We conducted a cohort study to assess risk factors for mortality in cases of ESBL-E bacteremia (*K*. *pneumoniae* or *E*. *coli*) and the risk factors for sepsis with organ failure. All consecutive patients of our institution from 2008 to 2011 with bacteremia due to ESBL-E were included. Basic epidemiological data, underlying comorbidities, origin of bacteremia, severity of sepsis and delay of appropriate anti-infective treatment were collected. Isolates were PCR-screened for the presence of ESBL genes and plasmid-mediated AmpC β-lactamases. Cox proportional hazard regression on mortality and multivariable logistic regression on risk factors for sepsis with organ failure was conducted. 219 cases were included in the analysis: 73.1% due to *E*. *coli*, 26.9% due to *K*. *pneumoniae*. There was no significant difference in hospital mortality (ESBL-E. coli, 23.8% vs. ESBL-*K*. *pneumoniae* 27.1%, p = 0.724). However, the risk of sepsis with organ failure was associated in cases of *K*. *pneumoniae* bacteremia (OR 4.5, p<0.001) and patients with liver disease (OR 3.4, p = 0.004) or renal disease (OR 6.8, p<0.001). We found significant differences in clinical presentation of ESBL-E bacteremia due to *K*. *pneumoniae* compared to *E*. *coli*. As *K*. *pneumoniae* cases showed a more serious clinical presentation as *E*. *coli* cases and were associated with different risk factors, treatment and prevention strategies should be adjusted accordingly.

## Introduction

Infections due to extended-spectrum beta-lactamase-producing *Enterobacteriaceae* (ESBL-E) are associated with impaired outcome compared to infections with susceptible pathogens [1–3]. Former studies on ESBL-E bacteremia proved that a delay of adequate antimicrobial chemotherapy can be an important factor on mortality [1, 2, 4]. This effect seems to be the most evident in cases of septic shock or organ failure [5]. This is even more important since there is evidence that infections due to *K*. *pneumoniae* are associated with a worse course compared to other *Enterobacteriaceae* [4, 6, 7]. However, studies concerning ESBL-E infections often do not differentiate between the infecting species. There are only few studies comparing outcome parameter of different *Enterobacteriaceae* [4, 6, 8–10]. To analyze the effect of the different ESBL-E species on mortality and on the clinical presentation, we conducted a cohort study comparing cases of ESBL-positive *K*. *pneumoniae-* and *E*. *coli* bacteremia including data on the timing of their antimicrobial treatment.

## Methods

### Study design

We conducted a retrospective cohort study on patients with bacteremia due to ESBL-E. The setting of this study was the Charité University Hospital in Berlin, Germany, a tertiary care university hospital with over 120,000 admissions per year. Previously, our ethics committee approved the study without informed consent. The patient data based on secondary clinical information. The biological material was obtained clinically and analyzed by a separate institution after anonymization in our institution (reference number EA4/031/11). Parts of the study have been published in different analyses including differing data sets [10, 11].

All inpatients with ESBL-E bacteremia (*E*. *coli* or K. *pneumoniae*) diagnosed between January 1^st^, 2008 and December 31^st^ 2011 were included. The bacteremia was classified as hospital onset if a positive blood culture was collected after the third hospital day. In case of more than one cultured organism in the first positive blood culture, the episode was defined as polymicrobial bacteremia. If the patient showed multiple subsequent positive blood cultures, the first positive blood culture lead to the allocation to one of the two pathogens and this episode was included in the analysis. Underlying comorbidities were assessed according to the method by Charlson et al. [12]. The comorbidities were collected using the patients’ ICD-10 codes and grouped for the calculation of the Charlson comorbidity index (CCI) according to the method by Thygesen et al. [13].

Further clinical parameters were collected by analysis of the patients’ files. To assess the origin of ESBL bacteremia we collected data on earlier infections during the analyzed hospital stay. These infections were due to same ESBL-positive organisms as the corresponding bacteremia episode and at maximum 14 days prior to the bacteremia onset. The infections were assessed according to the CDC definitions [14]. Primary bacteremia was defined as central venous catheter (CVC) <48h prior to the bacteremia onset without presence of another ESBL-E infection. Mortality was defined as in-hospital mortality and ESBL colonization was assessed as colonization with ESBL-E prior to the bacteremia episode at any site. Sepsis, severe sepsis, and septic shock were defined according to the definitions of the consensus conference of the American College of Chest Physicians and the Society of Critical Care Medicine which also find application in Germany [15, 16]. Delay of anti-infective treatment (DAT) was defined as initiation of effective treatment ≥1 day after onset of bacteremia. Effective treatment was defined as an antimicrobial agent the ESBL-positive organism was tested susceptible against.

### Microbiological methods

Species identification and antimicrobial susceptibility testing was performed using the Vitek 2 automated system with interpretation and antibiogram reporting according to the CLSI standard [17]. Confirmation of ESBL production was performed by a minimum inhibitory concentration dilution test on a multiwell microtiter plate. Three cephalosporins (cefotaxime, ceftazidime, cefpodoxime) were tested alone and in combination with ESBL inhibitor clavulanic acid. All verification swabs were inoculated onto chrome ID ESBL agar (BioMerieux). ESBL-positive isolates were affirmed by Double Disc Synergie Testing using 3rd generation cephalosporins with/without clavulanic acid (Mast). Species confirmation was done by API20E (BioMerieux). The isolates were screened for the presence of different ESBL genes (blaTEM-type, blaSHV-type, blaCTX-M-1/2/9 group) by polymerase chain reaction (PCR) and subsequent sequencing [18]. If none of these ESBL genes could be identified, additional PCR tests for the presence of plasmid-mediated AmpC β-lactamases [19] and further ESBL genes (blaCTX-M-8-type, blaCTX-M-26-type) were performed [20]. Furthermore, basic bacterial typing of all ESBL-positive E. coli isolates was performed by a PCR-based method for determination of the four major E. coli phylogenetic groups [21].

### Statistical methods

Parameters in the univariate analysis were tested using the Wilcoxon rank sum test for continuous variables and the Fisher’s exact test for categorical variables. For the analysis of factors associated to severity of illness, the categories were transformed to a binary category as bacteremia (bacteremia and sepsis) vs. sepsis with organ failure (severe sepsis and septic shock). Clinical parameter of *E*. *coli*- and *K*. *pneumoniae*- cases were compared using univariate analysis. Multivariable binary logistic analysis using a stepwise forward regression was computed for the analysis of clinical risk factors for sepsis with organ failure. We compared the clinical parameter of the deceased patients and the alive discharged patients using univariate analysis. We calculated adjusted hazard ratios for in-hospital death using Cox-proportional hazard regression using a stepwise forward approach. In both multivariable analyses, parameters with p-values <0.20 in the univariate analyses were considered in the analysis. Variables with p-values <0.05 were included and variables with p≥0.05 were excluded. All tests of were two tailed with a p-value <0.05 considered to be significant. Data was analyzed using IBM SPSS Statistics Version 22.

## Results

**Fig 1:**
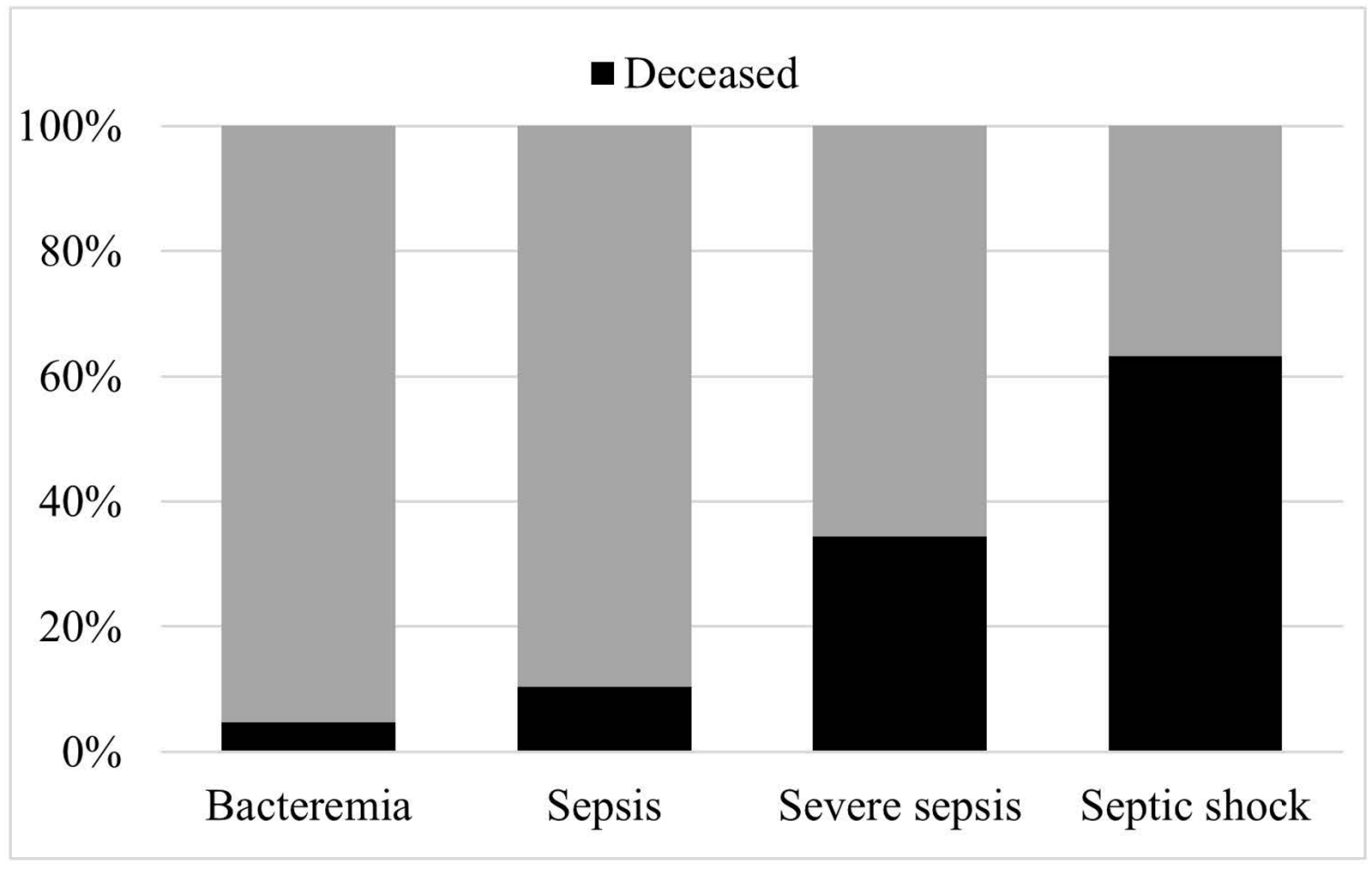
Severity of sepsis in relation to mortality rate.

We identified altogether 243 consecutive cases of bacteremia, 177 cases due to ESBL-positive *E*. *coli* (73.1%) and 66 cases due to *K*. *pneumoniae* (27.2%) together. No case showed both analyzed pathogens in the same blood culture. From twenty-three cases (9.9%), sufficient data was not available and therefore excluded. The remaining 219 cases were analyzed (n = 160, 73.1% due to *E*. *coli* and n = 59, 26.9% due to *K*. *pneumoniae*). We found an increasing risk for mortality in relation to the applied definitions for severity of sepsis (Fig 1). The characteristics of the analyzed cohort stratified by infecting organism are given in S1 Table. Patients suffering from ESBL-positive *K*. *pneumoniae* bacteremia (ESBL-KP-Bac) were younger than the compared patients with ESBL-positive *E*. *coli* bacteremia (ESBL-EC-Bac). They more often had a secondary bacteremia due to a surgical site infection and more often an unknown source of infection. ESBL-KP-Bac was more often associated with sepsis with organ failure. Patients with sepsis with organ failure showed significantly reduced DAT compared to patients presenting with bacteremia only (Median 0 days, IQR 0;2 days vs. Median 2 days, IQR 0;3 days, p = 0.003). Patients with ESBL-KP-Bac showed an increased mortality compared to ESBL-EC-Bac (27.1% vs. 23.8%) but not statistical significant. ESBL-EC-Bac however, was more common among patients with previous urinary tract infection. The mostly used antimicrobial agents after infection onset were carbapenems: 71.2% (n = 156), quinolones 12.3% (n = 27), tigecyclin 5.0% (n = 11), piperacillin-tazobactam 2.3% (n = 5) and gentamicin 2.3% (n = 5).

### Microbiology parameter

In the clinically reported antibiogram, 98.6% (n = 216) isolates were resistant to piperacillin/ tazobactam, 98.2% (n = 215) to ceftazidime, 69.4% (n = 152) to ciprofloxacin and 43.4% (n = 95) to gentamicin. None of the included isolates was reported resistant to the carbapenems imipenem or meropenem. Of the 219 isolates, 88.5% (n = 194) were available for further ESBL genotype analysis; the remaining 25 were not retrievable. The distribution of the ESBL genotypes is shown in Tables 1 and 2 with overall CTX-M-15, CTX-M-1, CTX-M-14 and SHV-5 as most common types. One-hundred and seven isolates (55.2%) carried two or more TEM- or SHV-type beta-lactamases. Seven isolates (3.6%) did not carry an ESBL gene. Five showed either TEM-181, TEM-1 or SHV-1 overproduction, one was CMY-positive and one did not show any beta-lactamase at all. The distribution pattern of the phylogenetic groups within the analyzed 140 *E*. *coli* isolates was B2 (33.6%, n = 47), A (28.6%, n = 40), D (26.4%, n = 37) and B1 (11.4%, n = 16).

**Table 1:** Univariate analysis of clinical parameter in patients presenting with sepsis with organ failure and bacteremia.

Parameter	Bacteremia (n = 138)	Sepsis with organ failure (n = 81)	P-value
Age years / Age < 61 years	67 (48.6%)	42 (51.9%)	0.676
Male sex	93 (67.4%)	53 (65.4%)	0.769
Charlson comorbidity index > 6	63 (45.7%)	51 (63.0%)	0.017
In-hospital death	12 (8.7%)	42 (51.9%)	<0.001
Polymicrobial bacteraemia	19 (13.8%)	10 (12.3%)	0.839
Hospital onset	74 (53.6%)	59 (72.8%)	0.006*
ESBL colonization before onset	85 (61.6%)	53 (65.4%)	0.664
Bacteraemia due to *E*. *coli*	115 (83.3%)	45 (55.6%)	<0.001*
Bacteraemia due to *K*. *pneumoniae*	23 (16.7%)	36 (44.4%)	
**Origin of ESBL-E bacteraemia**			
Urinary tract infection	55 (39.9%)	20 (24.7%)	0.027*
Lower respiratory tract infection	22 (15.9%)	18 (22.2%)	0.279
Intra-abdominal infection	9 (6.5%)	13 (16.0%)	0.035*
Surgical site infection	4 (2.9%)	4 (4.9%)	0.472
Primary bacteraemia	19 (13.8%)	11 (13.6%)	1.000
Other	6 (4.3%)	2 (2.5%)	0.756
Unknown Origin	35 (25.4%)	19 (23.4%)	0.883
**Severity of illness and delay of anti-infective treatment**			
Delayed adequate anti-infective treatment	70 (50.7%)	25 (30.9%)	<0.001
**ESBL Genotype**			
No ESBL genotype	4 (2.9%)	3 (3.7%)	0.711
CTX-M-1	30 (21.7%)	9 (11.1%)	0.066*
CTX-M-14	8 (5.8%)	6 (7.4%)	0.776
CTX-M-15	60 (43.5%)	38 (46.9%)	0.674
CTX-M-2	1 (0.7%)	-	1.000
CTX-M-2/97	2 (1.4%)	-	0.532
CTX-M-3	4 (2.9%)	2 (2.5%)	1.000
CTX-M-32	-	2 (2.5%)	0.136
CTX-M-55	1 (0.7%)	1 (1.2%)	1.000
CTX-M-61	1 (0.7%)	-	1.000
SHV-12	2 (1.4%)	-	0.532
SHV-2	1 (0.7%)	-	1.000
SHV-5	4 (2.9%)	9 (11.1%)	0.018*
SHV-7	-	1 (1.2%)	0.370
TEM-12	1 (0.7%)	-	1.000
TEM-52	3 (2.2%)	1 (1.2%)	1.000
Unknown (not available for genotype analysis)	16 (11.6%)	9 (11.1%)	1.000
**Underlying conditions**			
Heart disease	16 (11.6%)	23 (28.4%)	0.003*
Vascular disease	24 (17.4%)	21 (25.9%)	0.166*
Neurologic disease	14 (10.1%)	7 (8.6%)	0.815
Chronic pulmonary disease	17 (12.3%)	19 (23.5%)	0.038*
Connective tissue disease	3 (2.2%)	-	0.298
Ulcer disease	6 (4.3%)	2 (2.5%)	0.713
Liver disease	16 (11.6%)	25 (30.9%)	<0.001*
Diabetes mellitus	34 (24.6%)	20 (24.7%)	1.000
Moderate to severe renal disease	44 (31.9%)	62 (76.5%)	<0.001*
Cancer/immunological disease	63 (45.7%)	24 (29.6%)	0.022*

Continuous parameter are displayed as median (interquartile range), categorical parameter as number (percentage). ESBL, extended-spectrum beta-lactamase.

*, parameter was included in the multivariable analysis on risk factors for severe sepsis.

**Table 2:** Univariate analysis of survivors and non-survivors after ESBL-E sepsis

Univariate analysis			
Parameter	Survived (n = 165)	Deceased (n = 54)	P-value
Age years / Age < 61 years	80 (48.5%)	29 (53.7%)	0.534
Male sex	107 (64.8%)	39 (72.2%)	0.406
Charlson comorbidity index > 6	5 (3; 8)	8 (6; 10)	<0.001
Days from admission to onset	5 (1; 18)	27 (15; 24)	<0.001
Days from onset to discharge/death	14 (9; 26)	12 (2; 31)	0.504
Polymicrobial bacteremia	23 (13.9%)	6 (11.1%)	0.817
Hospital onset	89 (53.9%)	44 (81.5%)	<0.001*
ESBL colonization before onset	106 (64.2%)	32 (59.3%)	0.520
Bacteraemia due to *E*. *coli*	122 (73.9%)	38 (70.4%)	0.601
Bacteraemia due to *K*. *pneumoniae*	43 (26.1%)	16 (29.6%)	
**Origin of ESBL-E bacteraemia**			
Urinary tract infection	66 (40.0%)	9 (16.7%)	0.002*
Lower respiratory tract infection	24 (14.5%)	16 (29.6%)	0.024*
Intra-abdominal infection	11 (6.7%)	11 (20.4%)	0.007*
Surgical site infection	4 (2.4%)	4 (7.4%)	0.105*
Primary bacteraemia	10 (6.0%)	20 (37.0%)	<0.001*
Other	8 (4.8%)	-	0.199
Unknown Origin	44 (26.7%)	12 (22.2%)	0.647
**Severity of illness and delay of anti-infective treatment**			
Bacteremia/ sepsis	126 (76.4%)	12 (22.2%)	<0.001*
Severe sepsis/ septic shock	39 (23.6%)	42 (77.8%)	
Delayed anti-infective treatment (days)	1 (0;3)	1 (0;2)	0.028*
**ESBL Genotype**			
No ESBL genotype	6 (3.6%)	1 (1.9%)	1.000
CTX-M-1	31 (18.8%)	8 (14.8%)	0.682
CTX-M-14	11 (6.7%)	3 (5.6%)	1.000
CTX-M-15	71 (43.0%)	27 (50.0%)	0.431
CTX-M-2	1 (0.6%)	-	1.000
CTX-M-2/97	2 (1.2%)	-	1.000
CTX-M-3	6 (3.6%)	-	0.340
CTX-M-32	-	2 (3.7%)	0.060
CTX-M-55	1 (0.6%)	1 (1.9%)	0.433
CTX-M-61	1 (0.6%)	-	1.000
SHV-12	2 (1.2%)	-	1.000
SHV-2	1 (0.6%)	-	1.000
SHV-5	9 (5.5%)	4 (7.4%)	0.740
SHV-7	1 (0.6%)	-	1.000
TEM-12	1 (0.6%)	-	1.000
TEM-52	3 (1.8%)	1 (1.9%)	1.000
Unknown (not available for genotype analysis)	18 (10.9%)	7 (13.0%)	0.631
**Underlying conditions**			
Heart disease	19 (11.5%)	20 (37%)	>0.001*
Vascular disease	30 (18.2%)	15 (27.8%)	0.173*
Neurologic disease	18 (10.9%)	3 (5.6%)	0.299
Chronic pulmonary disease	24 (14.5%)	12 (22.2%)	0.206
Connective tissue disease	3 (1.8%)	-	1.000
Ulcer disease	5 (3.0%)	3 (5.6%)	0.411
Liver disease	20 (12.1%)	21 (38.9%)	>0.001*
Diabetes mellitus	36 (21.8%)	18 (33.3%)	0.103*
Moderate to severe renal disease	61 (37.0%)	45 (83.3%)	>0.001*
Cancer/immunological disease	66 (40.0%)	21 (38.9%)	1.000

Continuous parameter are displayed as median (interquartile range), categorical parameter as number (percentage). ESBL, extended-spectrum beta-lactamase.

*, parameter was included in the Cox regression analysis on risk factors for death.

### Factors associated with sepsis with organ failure

Table 3 shows the results of the multivariable analysis on risk factors for sepsis with organ failure. The *K*. *pneumoniae* cases were associated with 4.5 times higher odds for an organ failure at presentation. Furthermore, sepsis with organ failure was associated with liver disease (OR 3.3) and moderate to severe renal disease (OR 6.835).

**Table 3:** Results of the multivariable binary logistic regression analysis on risk factors for sepsis with organ failure

Parameter	P-value	OR	Upper—Lower CI 95
Moderate to severe renal disease	<0.001	6.835	3,485–13,405
Liver disease	0.004	3.347	1,463–7,658
Bacteremia due to *E*.*coli*	<0.001	1 = reference	
Bacteremia due to *K*. *pneumoniae*		4.499	2,168–9,337

OR, odds ratio. CI 95, 95% confidence interval.

### Factors associated with in-hospital mortality (Cox-proportional hazard analysis)

**Fig 2:**
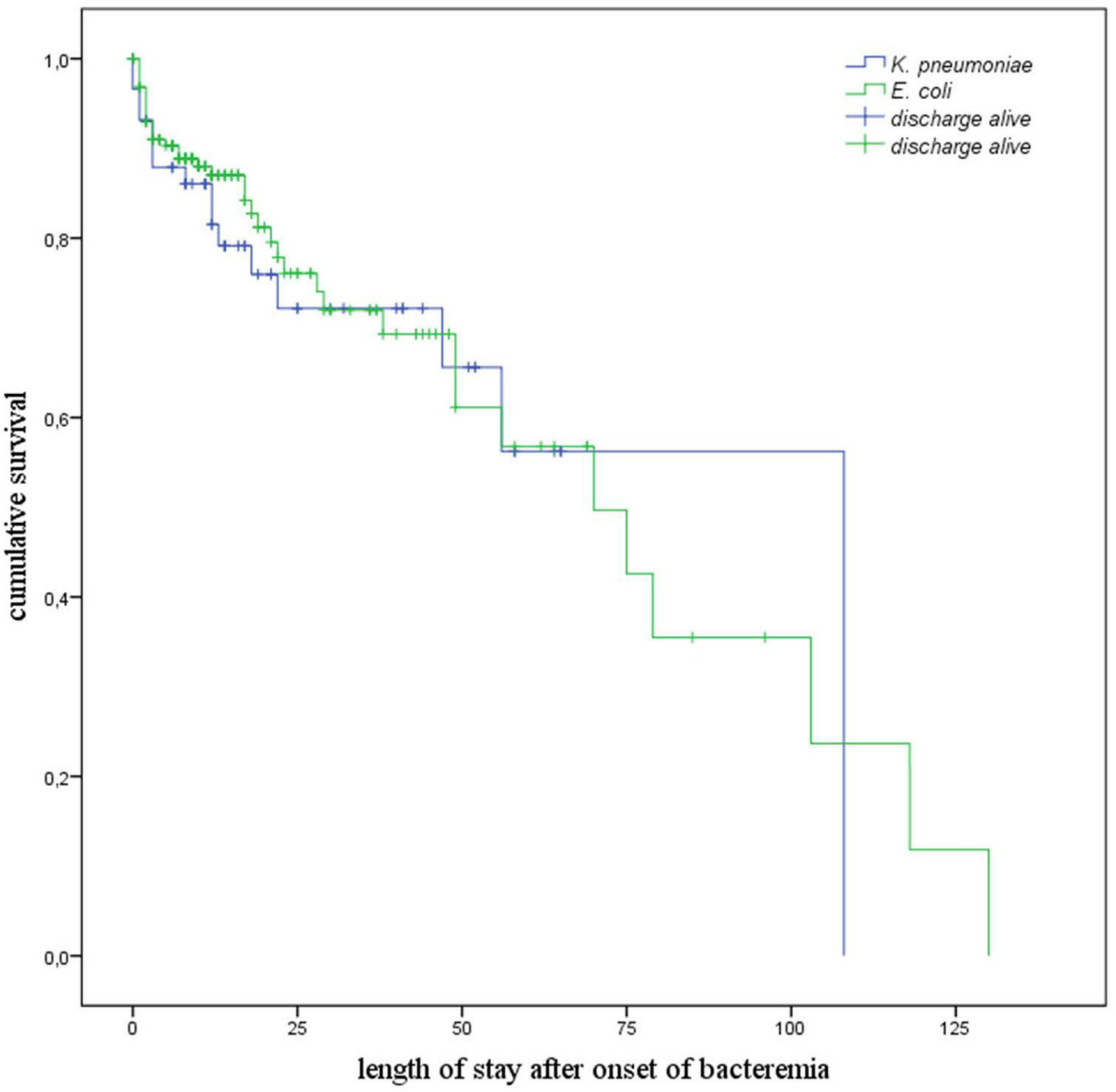
Kaplan Meier plot for cumulative survival associated with length of stay after onset of bacteremia stratified by infecting organism ESBL-positive -*E*. *coli* vs. -*K*. *pneumoniae*.

The results of the univariate analysis on in-hospital mortality are displayed in Table 2. In order to assess the effect of the infecting organism (*E*. *coli* vs. *K*. *pneumoniae*) on mortality this parameter was also considered in the Cox-proportional hazard regression. The deceased had significantly higher CCIs. These patients also had significantly more often a hospital onset bacteremia, sepsis with organ failure, prior episodes of lower respiratory tract infection, intra-abdominal infection or primary bacteremia, underlying heart disease, liver- or renal disease. The survivors had more commonly a urinary tract infection prior to their bacteremia. The results of the Cox-proportional hazard regression (Table 4) showed that sepsis with organ failure was associated with a 4.5-fold higher hazard for mortality, renal disease and liver disease with 2.7-fold and 1.8-fold elevated hazard. The only protective factor was urinary tract infection that was associated with a hazard reduction for mortality of about 61% (HR 0.39). There was no statistically significant difference in the mortality risk between both species. Fig 2 shows the Kaplan Meier plot for cumulative survival stratified by ESBL-E species in relation to length of stay after onset of sepsis.

**Table 4:** Results of the Cox-proportional hazard regression on clinical risk factors for death after ESBL-E sepsis.

Parameter	P-value	HR	Upper—Lower CI 95
Bacteremia due to *E*.*coli*	0.060	1 = reference	
Bacteremia due to *K*. *pneumoniae*		1.801	0.975–3.330
Urinary tract infection	0.007	0.360	0.172–0.754
Bacteremia/sepsis	<0.001	1 = reference	
Severe sepsis/ septic shock		4.543	2.134–9.673
Liver disease	0.045	1.801	1.013–3.203
Moderate/ severe renal disease	0.016	2.675	1.198–5.973

HR, hazard ratio. CI 95, 95% confidence interval.

## Discussion

In this study ESBL-KP-Bac bacteremia was associated with different origin, with sepsis with organ failure and younger age compared to ESBL-EC-Bac cases (S1 Table). Our results underline the findings of previous studies showing that *K*. *pneumoniae* infections are associated with more serious illness than *E*. *coli* infections [4, 6, 7, 22]. Even though *K*. *pneumoniae* bacteremia was not associated with delayed adequate anti-infective treatment it was associated with slightly increased mortality, but it did not reach the significance level. The small sub-cohort of ESBL-KP-Bac might explain this.

Several studies show that a delay of adequate antimicrobial chemotherapy is a risk factor for in-hospital death [1, 2, 4]. In our cohort, the association of sepsis with organ failure and reduced DAT demonstrate most likely the realization of the German sepsis guidelines [23]. Patients who present with severe sepsis or septic shock are recommended to be immediately initiated with an early initial broad-spectrum treatment including reserve antibiotics e.g. carbapenems. In our study, the presentation of severe sepsis happened before the initiation of the antimicrobial therapy. Hence, the observed association between disease severity and reduced DAT represents the response to the severity of the disease and not the cause of the disease. This refers to the observation that in sepsis with organ failure, an early effective antimicrobial therapy is associated with significantly reduced mortality risk [24]. Even though most cases of sepsis with organ failure received appropriate antimicrobial therapy within the first day, more than 25% showed a DAT of more than 1 day. This observation might at least partly explain the remaining high mortality (51.9%) in patients with severe sepsis and septic shock.

ESBL-KP-Bac cases were associated with sepsis with organ failure. However, they did not show significant differences in comorbidities compared to ESBL-EC-Bac cases (S1 Table). This might be explained by a potentially higher virulence of *K*. *pneumoniae* compared to *E*. *coli*. An earlier study on length of hospital stay included parts of the data at hand. Altogether 1.851 cases of bacteremia with (ESBL-positive and–negative) *Enterobacteriaceae* were analyzed then [10]. In that study, *K*. *pneumoniae* cases were associated with significantly prolonged hospital stay compared to *E*. *coli* cases. This most likely indicates a more problematic course of infection in *K*. *pneumoniae* cases. However, in that former study no data on antimicrobial therapy was analyzed [10]. Our present results support the previous findings after adjustment for timely and adequate antimicrobial therapy.

The primary source of bacteremia differed significantly between both pathogens. While *E*. *coli* bacteremia was mostly found secondary to a urinary tract infection, *K*. *pneumoniae* cases were associated with surgical site infection (SSI), lower respiratory tract infection (LRTI) and unknown origin. Besides undetected colonization, the latter could be explained by health-care associated transmission. However, all sites of origin (SSI, LRTI and transmission) are likely since *K*. *pneumoniae* is often identified as outbreak pathogen [25, 26] and shows higher transmission potential than *E*. *coli* [27].

Urinary tract infection (UTI) as possible source of bacteremia was found less often associated with the development of a fatal bacteremia. This goes along with former studies on ESBL-E bacteremia [7, 28]. ESBL-E are common pathogens of urinary tract infections. A prior microbiologically diagnosed ESBL-E UTI might have supported an early initiation of anti-infective treatment at presentation of a secondary bacteremia.

Even though there are significant differences in the ESBL genotype distribution of both pathogens, none of these genotypes was associated with increased mortality. However, our data confirms that the most common genotypes among clinical ESBL-positive *E*. *coli* isolates in the United States and in Europe are currently CTX-M-15 and CTX-M-14 [29, 30]. In our *K*. *pneumoniae* isolates the most common ESBL genotypes were CTX-M-15 and SHV-5 which is also commonly observed in Europe [3, 31].

In our study, most of the isolates were reported resistant against piperacillin-tazobactam (pip-taz) due to their ESBL positivity. In 2011, CLSI recommended the interpretation of the breakpoint should be reported as found, irrespective of whether there was ESBL production [32]. Based on the current CLSI breakpoints, 35.2% of our isolates would be resistant to pip-taz. However, in this study, we focused on the results of the treatment based on the reported antibiogram. This goes along with the observed antimicrobial treatment showing carbapenems as mostly used agent, followed by quinolones.

This study has limitations. It was performed retrospectively and only ESBL-positive infections were assessed. Potential differences to ESBL-negative infections cannot be determined. The study was conducted at a single center. However, the data were collected from all patients within our hospital, regardless the respective department and likely represents the current course of these kind of infections in comparable hospitals. The species identification was performed using the Vitek 2 system. Since Vitek 2 cannot differentiate *K*. *pneumoniae* and *K*. *variicola*, it is possible that up to 10% of our analyzed *K*. *pneumoniae* isolates are *K*. *variicola*. Future studies on carbapenem resistance should include ertapenem. Some isolates produce weak carbapenemases and may show decreased susceptibility to this substance only.

In conclusion, ESBL-positive *K*. *pneumoniae* bacteremia compared to ESBL-positive *E*. *coli* bacteremia is often associated with complicated infection and less often with uncomplicated infection such as urinary tract infection. In this small study group, pathogen on species level and genotypes were not associated with mortality, but with well-known factors as sepsis with organ failure. Knowledge of colonization and source of infection should be considered for empiric anti-infective treatment, especially in patients with septic shock to reduce DAT and mortality. Infections with ESBL-positive *K*. *pneumoniae* should be considered as more serious infections than comparable *E*. *coli* infections. Treatment and prevention strategies should be adjusted accordingly.

## Supporting Information

S1 Table Univariate analysis of clinical parameter in patients with ESBL-positive bacteremia due to *E*. *coli* in comparison to *K*. *pneumoniae*. Continuous parameter are displayed as median (interquartile range), categorical parameter as number (percentage). ESBL, extended-spectrum beta-lactamase.
(DOCX)

S2 Table Raw data. (XLS)
