# Supplementary material for: Sepsis Caused by Extended-Spectrum Beta-Lactamase (ESBL)-Positive K. pneumoniae and E. coli: Comparison of Severity of Sepsis, Delay of Anti-Infective Therapy and ESBL Genotype
Source: PLoS One. 2016 Jul 21;11(7):e0158039. doi: 10.1371/journal.pone.0158039 (PMC4956035; doi:10.1371/journal.pone.0158039)
Supplement: S1 Table — Continuous parameter are displayed as median (interquartile range), categorical parameter as number (percentage). ESBL, extended-spectrum beta-lactamase. (DOCX) [file pone.0158039.s001.docx]

S1 Table. Univariate analysis of clinical parameter in patients with ESBL-positive bacteremia due to *E. coli* in comparison to *K. pneumoniae.*

| **Univariate analysis** | | | | | | | | |
| --- | --- | --- | --- | --- | --- | --- | --- | --- |
| **Parameter** | **E. coli**  **(n=160)** | | **K. pneumoniae (n=59)** | | **P-value** | | | |
| Age years / Age < 61 years | 72 (45.0%) | | 37 (62.7%) | | 0.023 | | | |
| Male sex | 105 (65.6%) | | 41 (69.5%) | | 0.631 | | | |
| Charlson comorbidity index (CCI) | 5 (3; 9) | | 6 (4; 8) | | 0.197 | | | |
| In-hospital death | 38 (23.8%) | | 16 (27.1%) | | 0.724 | | | |
| Polymicrobial bacteraemia | 22 (13.8%) | | 7 (11.9%) | | 0.825 | | | |
| Days from admission to onset | 6 (1; 21) | | 19 (2; 52) | | 0.005 | | | |
| Days from onset to discharge/death | 14 (8; 40) | | 12 (7; 25) | | 0.300 | | | |
| Hospital onset | 92 (57.5%) | | 41 (69.5%) | | 0.120 | | | |
| ESBL colonization before onset | 106 (66.3%) | | 32 (54.2%) | | 0.116 | | | |
| **Origin of ESBL-E bacteraemia** |  | |  | |  | | | |
| Urinary tract infection | 63 (39.4%) | | 12 (20.0%) | | 0.010 | | | |
| Lower respiratory tract infection | 25 (15.6%) | | 15 (25.4%) | | 0.115 | | | |
| Intra-abdominal infection | 17 (10.6%) | | 5 (8.5%) | | 0.802 | | | |
| Surgical site infection | 3 (1.9%) | | 5 (8.5%) | | 0.035 | | | |
| Primary bacteraemia | 25 (15.6%) | | 5 (8.5%) | | 0.249 | | | |
| Other | 8 (5.0%) | | - | | 0.154 | | | |
| Unknown origin | 31 (19.4%) | | 23 (40.0%) | | 0.006 | | | |
| **Severity of sepsis and delay of anti-infective therapy** | |  | |  | |  |  |  |
| Bacteremia/ sepsis | 115 (71.9%) | | 23 (39.0%) | | <0.001 | | | |
| Severe sepsis/ septic shock | 45 (28.1%) | | 36 (61.0%) | |  |  |  |  |
| Delayed anti-infective treatment (days) | 1 (0;3) | | 0 (0;2) | | 0.116 | | | |
| **ESBL Genotype** |  | |  | |  | | | |
| No ESBL genotype | 4 (2.5%) | | 3 (5.1%) | | 0.390 | | | |
| CTX-M-1 | 38 (23.8%) | | 1 (1.7%) | | <0.001 | | | |
| CTX-M-14 | 13 (8.1%) | | 1 (1.7%) | | 0.119 | | | |
| CTX-M-15 | 68 (42.5%) | | 30 (50.8%) | | 0.287 | | | |
| CTX-M-2 | 1 (0.6%) | | - | | 1.000 | | | |
| CTX-M-2/97 | 2 (1.3%) | | - | | 1.000 | | | |
| CTX-M-3 | 3 (1.9%) | | 3 (5.1%) | | 0.347 | | | |
| CTX-M-32 | 2 (1.3%) | | - | | 1.000 | | | |
| CTX-M-55 | 1 (0.6%) | | 1 (1.7%) | | 0.467 | | | |
| CTX-M-61 | 1 (0.6%) | | - | | 1.000 | | | |
| SHV-12 | 2 (1.3%) | | - | | 1.000 | | | |
| SHV-2 | - | | 1 (1.7%) | | 0.269 | | | |
| SHV-5 | - | | 13 (22%) | | <0.001 | | | |
| SHV-7 | - | | 1 (1.7%) | | 0.269 | | | |
| TEM-12 | 1 (0.6%) | | - | | 1.000 | | | |
| TEM-52 | 4 (2.5%) | | - | | 0.576 | | | |
| Unknown (not available for genotype analysis) | 20 (12.5%) | | 5 (8.5%) | | 0.481 | | | |
| **Underlying conditions** |  | |  | |  | | | |
| Heart disease | 27 (16.9%) | | 12 (20.3%) | | 0.555 | | | |
| Vascular disease | 36 (22.5%) | | 9 (15.3%) | | 0.264 | | | |
| Neurologic disease | 14 (8.8%) | | 7 (11.9%) | | 0.605 | | | |
| Chronic pulmonary disease | 22 (13.8%) | | 14 (23.7%) | | 0.099 | | | |
| Connective tissue disease | 3 (1.9%) | | - | | 0.565 | | | |
| Ulcer disease | 7 (4.4%) | | 1 (1.7%) | | 0.686 | | | |
| Liver disease | 29 (18.1%) | | 12 (20.3%) | | 0.700 | | | |
| Diabetes mellitus | 37 (23.1%) | | 17 (28.8%) | | 0.383 | | | |
| Moderate/ severe renal disease | 72 (45.0%) | | 34 (57.6%) | | 0.127 | | | |
| Cancer/ immunological disease | 65 (40.6%) | | 22 (37.3%) | | 0.756 | | | |

Continuous parameter are displayed as median (interquartile range), categorical parameter as number (percentage). ESBL, extended-spectrum beta-lactamase.
